# The Four‐Square Step Test With and Without Dual Tasks Among Older Adults With and Without a Fall History: A Retrospective Cohort Study

**DOI:** 10.1002/hsr2.71820

**Published:** 2026-02-13

**Authors:** Mario Baker, Liana Burk, Meredith Gardner

**Affiliations:** ^1^ Department of Physical Therapy The University of Findlay Findlay Ohio USA

**Keywords:** falls, four‐square step test, older adults

## Abstract

**Background and Aim:**

As the global population of adults aged 65 and older increases, so does the prevalence of falls, which are the second leading cause of unintentional injury deaths worldwide, according to the World Health Organization. The Four‐Square Step Test (FSST) is a widely used tool for assessing dynamic balance and mobility. The purpose of this study was to compare the use of the FSST and the FSST with dual task(s) (DT) for discriminating between older adult fallers and non‐fallers by sex.

**Methods:**

Fifty community‐dwelling older adults (35 females, 15 males) participated. Based on self‐reported fall history, 28 were classified as non‐fallers and 22 as fallers. Participants completed the FSST, FSST with a motor DT, and FSST with a cognitive DT. The area under the curve (AUC) of the receiver operating characteristic curve was calculated and used to compare the FSST conditions to determine the best condition among the three used for discriminating between faller and non‐faller participants.

**Results:**

Among females, AUCs were 0.634 (FSST), 0.690 (motor DT), and 0.668 (cognitive DT). Among males, AUCs were 0.518 (FSST), 0.518 (motor DT), and 0.732 (cognitive DT). For all participants, cutoff scores for distinguishing fallers from non‐fallers were approximately 10.07 s (FSST), 10.05 s (motor DT), and 13.22 s (cognitive DT).

**Conclusion:**

The FSST with a cognitive DT showed the highest overall accuracy in distinguishing fallers, particularly among males. The FSST with a motor DT demonstrated better discriminative performance than the standard FSST in females, while both test conditions were equivalent in males. Identified cutoff scores were approximately 13 s for the cognitive dual task and 10 s for both the standard and motor dual task conditions. These findings support the potential clinical utility of incorporating cognitive or motor dual tasks into the FSST, particularly for sex‐specific fall risk assessment in community‐dwelling older adults.

## Summary

- The clinical motivation for this study was to identify an alternative balance assessment for physical therapists to use in the identification of older adult fallers.
- In general, the Four‐square step test (FSST) with motor and cognitive dual tasks (DT) was more accurate in the identification of individuals with a fall history when compared to the FSST without DT.
- In the older adult population, the FSST with dual tasks could be used as an alternative assessment for discriminating between community‐dwelling older adult fallers and non‐fallers.

## 1 Introduction

As the global population of individuals aged 65 years and older continues to grow [1], the prevalence of falls is rising significantly both in the United States and worldwide. In 2019 alone, falls among adults aged 65 and older accounted for approximately 34,000 deaths and 3 million emergency department visits in the United States [2]. Globally, the World Health Organization (WHO) ranks falls as the second leading cause of unintentional injury deaths, with adults over 60 experiencing the highest rates of fatal falls [3]. In the United States, 20% to 30% of older adults who fall sustain injuries such as bruises, hip fractures, or head trauma [3].

Early identification of individuals at risk for falls is a critical step in fall prevention [4]. Effective prevention strategies depend on the use of valid and reliable tools to assess balance and mobility [4]. Evidence consistently indicates that fall risk increases with age, partly due to age‐related declines in physical, sensory, and cognitive functions [3]. Furthermore, a prior history of falls is one of the strongest predictors of future falls [5].

The Four‐Square Step Test (FSST) is a performance‐based clinical assessment tool designed to evaluate dynamic balance and fall risk in older adults [6]. The FSST demonstrates excellent test‐retest and inter‐rater reliability, with intraclass correlation coefficients (ICC) of 0.98 and 0.99, respectively [6]. It also shows strong concurrent validity when compared with other established measures such as the Step Test and the Timed Up and Go Test [6]. The FSST requires individuals to step in multiple directions over low obstacles, and the test can be completed in under 5 min, making it a practical and efficient option for clinical settings [4]. It has been validated in various populations, including older adults with and without a history of falling [4]. For example, Mathurapongsakul and Siriphorn reported a FSST cutoff score of 10.14 s, with both sensitivity and specificity at 66.7% when distinguishing between fallers and non‐fallers in the older adult population [4].

Emerging evidence highlights the role of cognition in balance and fall risk. [7] Even subtle cognitive changes in the absence of dementia have been associated with increased fall risk in older adults [7, 8, 9]. Given that most activities of daily living (ADLs) require simultaneous motor and cognitive processing, incorporating dual‐tasks into balance assessments may enhance their predictive utility [7]. Therefore, there is a growing need for brief, performance‐based assessments that integrate both motor and cognitive components to better identify fall risk in the geriatric population.

To date, no published study has directly compared the standard FSST to a dual‐task (DT) version (FSST‐DT) in the geriatric population. The discriminative ability of the FSST when combined with cognitive and motor dual tasks remains underexplored. The primary aim of this study was to compare the effectiveness of the FSST and the FSST‐DT in distinguishing between older adult fallers and non‐fallers, in order to determine which version more accurately identifies individuals at risk. A secondary aim was to examine whether performance differed by sex. We hypothesized that the FSST‐DT would better discriminate individuals with a history of falling within the past year than the standard FSST, regardless of sex.

## 2 Methods

### 2.1 Participants

This retrospective cohort study was approved by the Institutional Review Board (IRB) for research involving human subjects at the researching university's IRB (No. 1662). Sixty‐one participants were recruited and screened from a sample of convivence and 50 participants met the inclusion criteria. Demographics of the participants are presented in Table 1. The mean age of the participants was 74.10 years ± 9.036 with a minimum age of 60 years and a maximum age of 101.00 years. All participants identified as white and non‐Hispanic. Of the 50 participants, 70% (*n* = 35) were female and 30% (*n* = 15) were male. The number of falls within the past 12 months is presented in Table 1. The mean number of reported falls in the past twelve months for all participants was 0.760 ± 1.318 with a minimum number of falls at 0 and a maximum number of falls at 8. Given the incidence of self‐reported falls among geriatric community dwelling older adults is 25% [10], using an alpha of 0.05, beta of 0.2, and power of 0.8, a sample size of 44 participants would be necessary to adequately power this study for geriatric fallers and non‐fallers, but not for a sex‐specific analyses with only 15 males.

**Table 1:** Demographic information of participants.

	Age (*n* = 50)	Number of falls in the past 12 months	Race (*n* = 50)	Ethnicity (*n* = 50)	Gender (*n* = 50)
	—	—	White	Non‐Hispanic	Female (*n* = 35)
	—	—	—	—	Male (*n* = 15)
Mean	74.100	0.760			
Std. deviation	9.036	1.318			
Minimum	60.000	0.000			
Maximum	101.000	8.000			

### 2.2 Inclusion/Exclusion Criteria

The inclusion criteria were older adults greater than or equal to sixty (60) years; the ability to communicate and follow greater than or equal to three step commands; the ability to walk at least 20 feet with or without an assistive device; the ability to perform basic ADL without assistance (dressing, eating, functional mobility, personal hygiene/grooming, bathing, toileting, and ambulation); dwells in the community; and able to complete the Mini‐Mental State Examination (MMSE) with a score greater than or equal to 24/30. The exclusion criteria were a medical condition that would prevent full participation in balance testing; a medical condition that would prevent full participation in the cognitive testing; pending litigation; vestibular disorders; blurred vision; diplopia (double vision); musculoskeletal injury or disease that influences walking ability; neurological disease that impairs walking ability; Parkinson's disease; cerebral vascular accident with residual deficits that impair walking ability; and a dementia diagnosis.

### 2.3 Procedure

Participants gave informed consent for participation in this study. The participants that met the inclusion criteria were assessed using the FSST, FSST with motor DT (carrying a cup of water), and FSST with cognitive DT (reciting every other letter of the alphabet). The motor dual task of carrying a cup of water was selected based on a previous study by Brustio et al. in the geriatric population [11] and the use of reciting every other letter of the alphabet was based on a dual task study by Henning et al. [12]. Each participant was required to sign an informed consent document that educated the participants on their rights and outlined the specific details of the study. Individuals were not compensated for their participation. The participants were then taken through a screening interview using a questionnaire to determine their eligibility for participation. The Participant Screening Interview Questionnaire (see Appendix A) was used to determine an individual's eligibility for the study as well as collect general demographic information, including the participants' previous history of falls. Next, the MMSE was administered (see Appendix B). The MMSE is a tool that is utilized to assess an individual's cognition. A score of 24 or higher indicates normal cognitive levels or no cognitive impairment [13]. The MMSE is a valid measure of cognition and has a sensitivity of 88% and a specificity of 86.2% [13]. If a participant met all of the inclusion criteria, researchers then took the participants' vitals. This included heart rate, blood pressure, temperature and respiration rate. The FSST was administered by the same researcher for intra‐rater reliability as a stand‐alone test and then with the inclusion of a motor and a cognitive dual task. Between each test condition participants had the option to take a 1–2‐min break. At the end of the session, participants were given an explanation of their fall risk based on the results of the standardized FSST without DT.

### 2.4 Statistical Analysis

The data was analyzed using the Jamovi software, version 2.3.21, for statistical analysis. First, a descriptive statistics analysis was conducted to characterize the sample. Then, the area under the curve (AUC) of the receiver operating characteristic (ROC) curve was calculated and used to compare the FSST with and without DT to determine which test best discriminated between the faller and non‐faller participants. AUC values indicate the following: high accuracy when AUC > 0.9, moderate accuracy when AUC = 0.7–0.9, low accuracy when AUC = 0.5–0.69, and a value due to chance when AUC < 0.5 [4]. The cut‐off scores were determined using the data from the ROC curve analysis, which selected the highest sensitivity and specificity. A Pearson's Chi‐Squared test was also used to determine the accuracy of a 10 s cut off score in predicting fallers versus non‐fallers for the FSST without DT.

## 3 Results

### 3.1 Participant Characteristics

There were two groups of participants that included fallers and non‐fallers. The 28 individuals that reported no falls in the past year had an average age of 77 years and 75% (*n* = 21) were female and 25% (*n* = 7) were male. The 22 individuals that reported at least one fall in the past year had an average age of 77 years and 64% (*n* = 14) were female and 36% (*n* = 8) were male.

### 3.2 FSST Performance and ROC Analysis

**Figure 1:**
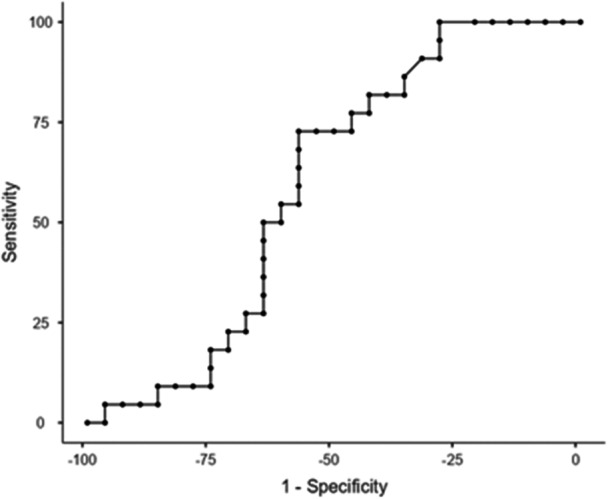
Receiver Operating Curve (ROC): FSST without Dual Task Time.

**Figure 2:**
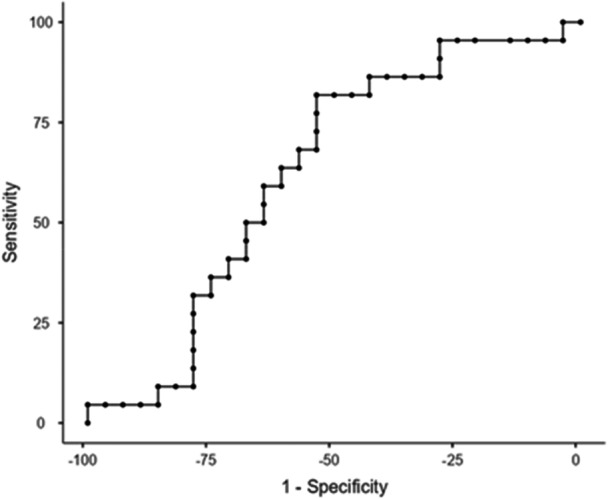
Receiver Operating Curve (ROC): FSST with Motor Task Time.

**Figure 3:**
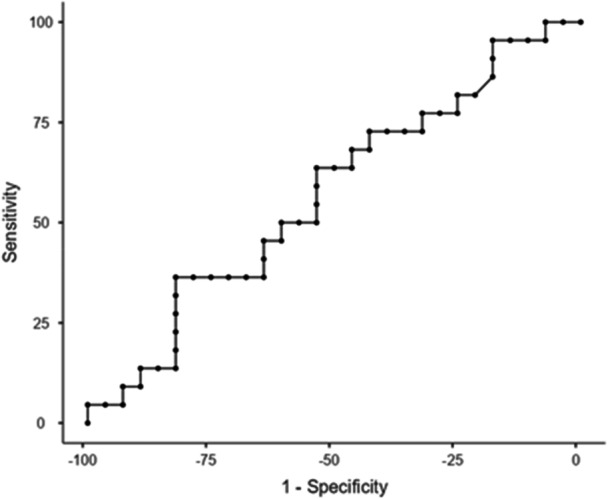
Receiver Operating Curve (ROC): FSST with Cognitive Task Time.

The percentiles of the FSST, FSST with cognitive DT, and FSST with motor DT of fallers and non‐fallers are presented in Table 2. The cut‐off score of the FSST without DT for all participants was 10.07 s with sensitivity (SN) = 72.73%, specificity (SP) = 57.14%, and the AUC = 0.590 (Table 3 and Figure 1). The cut‐off score of the FSST with motor DT for all participants was 10.05 s with a SN = 81.82%, SP = 53.57%, and AUC = 0.623 (Table 3 and Figure 2). The cut point of the FSST with cognitive DT for all participants was 13.22 s with SN = 72.73%, SP = 42.86%, and AUC = 0.569 (Table 3 and Figure 3). All AUCs fall in the 0.56 to 0.62 range, indicating low to fair accuracy for discriminating between fallers and non‐fallers. The FSST with motor DT shows the highest AUC (0.623), but the confidence intervals for all tests overlap, suggesting no statistically significant difference between them. The wide confidence intervals reflect a relatively small sample size or variability in test performance.

**Table 2:** FSST, FSST with Cognitive Task, and FSST with Motor Task Percentiles of Fallers and Non‐Fallers.

	FSST without dual task time, seconds		FSST with cognitive task time, seconds		FSST with motor task time, seconds	
	Non‐faller	Faller	Non‐faller	Faller	Non‐faller	Faller
*n*	28	22	28	22	28	22
25^th^ percentile	8.270	9.530	11.980	12.418	8.502	10.260
50^th^ percentile	9.615	10.835	13.730	14.280	9.720	11.255
75^th^ percentile	14.080	12.405	15.715	20.283	12.453	13.220
Standard deviation (SD)	17.952	6.070	8.222	9.315	8.142	10.243

**Table 3:** FSST without Dual Task, with Motor Dual Task, and with Cognitive Dual Task Cut Off Scores with Sensitivity, Specificity and the Area Under the Curve of the Receiver Operating, Curve.

Condition	Cut off score, seconds	Sensitivity (%)	Specificity (%)	AUC	95% Confidence interval
FSST without dual task	10.07	72.73%	57.14%	0.590	(0.428, 0.752)
FSST with motor task	10.05	81.82%	53.57%	0.623	(0.464, 0.783)
FSST with cognitive task	13.22	72.73%	42.86%	0.569	(0.406, 0.732)

### 3.3 FSST Performance and Chi Squared Analysis

A Pearson Chi Squared test was performed for the FSST without DT at a cut off score of 10 s, with a value of 4.4611, and probability equal to 0.035 indicating statistical significance for the FSST without a DT with the 10 s cut off score. This indicated that 72.73% of predicted non‐fallers on the FSST without DT didn't fall and 57.14% of predicted fallers did have a fall (Table 4). There was no statistically significant difference using the Pearson Chi Squared test for the FSST with motor or cognitive DT in this population of participants.

**Table 4:** Pearson's Chi‐Squared Test Results for the FSST without Dual Task for the 10 s Cut Off Score for all Participants.

FSST without Dual Task			
10 s cut off score determination	Self‐reported status		Total
	Non‐faller	Faller	
**Non‐faller**	16	6	22
Frequency	12.4	9.7	22
Expected frequency	72.73	27.27	100
Row percentage	57.14	27.27	44
**Faller**	12	16	28
Frequency	15.7	12.3	28
Expected frequency	42.86	57.14	100
Row percentage	42.86	72.73	56
**Total**	28	22	50
Frequency	28	22	50
Expected frequency	56	44	100
Row percentage	100	100	100

At a 10.25 s cutoff score, the FSST with cognitive DT for females resulted in a SN = 85.71%, SP = 61.9%, and AUC = 0.690 (Table 5). At a 13.85 s cut‐off score, the FSST with cognitive DT for females resulted in a SN = 71.43%, SP = 66.67%, and AUC = 0.668 (Table 5). At an approximate 17.8 s cut‐off score, the FSST with cognitive DT for males resulted in a SN = 28.57%, SP = 100%, and AUC = 0.518 (Table 5). At a 13.99 s cut‐off score, the FSST with motor DT for males resulted in a SN = 85.71%, SP = 62.5%, and AUC = 0.732 (Table 5). At a 15.20 s cut‐off score, the FSST with a motor DT for males resulted in a SN = 57.14%, SP = 87.5%, and AUC = 0.732 (Table 5).

**Table 5:** FSST with Cognitive Task and Motor Task by Sex (Male and Female).

	Cut off score, seconds	Sensitivity (%)	Specificity (%)	AUC
FSST Motor task female	9.32	92.86%	52.38%	0.690
	9.74	85.71%	57.14%	0.690
	10.25	85.71%	61.9%	0.690
FSST Cog task female	13.79	71.43%	61.9%	0.668
	13.85	71.43%	66.67%	0.668
	17.34	50%	85.71%	0.668
FSST Motor task male	17.8	28.57%	100%	0.518
FSST Cog task male	13.99	85.71%	62.5%	0.732
	15.12	57.14%	87.5%	0.732

## 4 Discussion

### 4.1 Comparison With Previous Research

Using the FSST with a DT as a predictor of fall risk in older adults does not frequently appear in the literature, but has been an area of interest within the past 7 years. Two scholarly projects by Haugeberg [14] in 2020 and Hoffman and Bucholz [15] in 2019 examined the relationship between completing the FSST with the addition of a cognitive DT. All participants in both studies, aged across the lifespan, completed multiple outcome measures and a cognitive screen before performing the FSST with and without a cognitive DT. Haugeberg concluded that the FSST is a means by which therapists can assess fall risk in community‐dwelling individuals, but recognized that more research is needed in order to establish the validity of their findings [14]. The study by Hoffman and Bucholz found that the times to complete the regular FSST and the cognitive FSST increased with each age group, indicating increased risk for falls [15]. They also found that older men had increased test times when performing a dual cognitive task with the FSST, whereas women had more consistent findings across age groups [15]. The overall conclusions of the studies indicated that the FSST is a test that can assess fall risk in community‐dwelling individuals, but more research in this area is needed to establish normative data for a cognitive FSST‐DT. Both studies recognized the ability of the FSST with additional cognitive challenges is good for assessing balance, making it an ideal activity to use for multi‐tasking to assess functional balance ability in community‐dwelling individuals.

A study organized by Erel in 2020 at Pamukkale University in Turkey includes 60 participants, all between the ages of 50–65 who were community dwelling individuals [16]. The purpose was to explain the motor and cognitive functions required when performing a FSST with DT in middle‐aged individuals [16]. The participants were to complete the FSST with no DT, a FSST with a motor dual task, and a FSST with a cognitive DT [16]. The article indicates that recruitment for the study ended, and no results of the study have been published since September of 2020. There is no evidence that the study was completed.

Finally, an article by Brustio et al. addressed a population of 60 older adults in order to assess the relationship between the effect of 16 weeks of DT training in older adults with reduced quality of life [11]. The mean age of the participants was 74.4 years, and all participants were tested with a 6‐min walk test, TUG test and FSST. DT participants were required to complete the above tests either while carrying a glass of water or while carrying a ball on a round tray, simulating a motor DT [11]. The results of the study suggested that 16 weeks of motor DT training, including motor manipulation of common everyday objects, could improve mobility in older adults [11].

### 4.2 Interpretation of Findings

In reviewing the existing literature on performing the FSST with DT, we found that the current literature does not solely focus on a population of community‐dwelling adults over the age of 60, but explored a wide age range of community‐dwelling adults participating in the FSST both with and without DT. Thus, the relevance of our study in the geriatric population was warranted.

The results of this study suggest the FSST with cognitive DT was more accurate than the FSST for discriminating between the faller and non‐faller older adult participants. In addition, the FSST with motor DT was more accurate than the FSST for discriminating between female fallers and non‐fallers, but was equal to the FSST when discriminating between male fallers and non‐fallers. The suggested cut off score for discriminating between non‐fallers and fallers was approximately 13 s for the FSST with a cognitive DT, 10 s for the FSST with a motor dual task, and 10 s for the FSST without a DT.

Evidence from this study suggests that with increased time to complete the standard FSST without DT, individuals are at an increased risk for falls [4, 6]. In addition, these same individuals are more susceptible than those who have the ability to perform the FSST with DT in a shorter period of time. Furthermore, evidence from this study suggests that individuals performing the FSST with a cognitive DT will need additional time to complete the FSST. Our results suggest that individuals who require more time to complete the FSST with DT were at an increased risk of falling. As a result, the FSST with DT was more indicative of falls than the FSST alone in this study.

### 4.3 Limitations

There were several limitations to consider regarding this study. First, participants were given the opportunity to practice the FSST prior to the test, and may have performed the tests conditions more efficiently on consecutive trials. Evidence of this can be found in the study as the data suggests that the individuals, on average, scored higher or completed the cognitive FSST faster in comparison to the standard FSST and the motor FSST. Secondly, we gathered a participant‐reported fall history. It is possible that participants did not report falls secondary to recall bias or did not completely understand the definition of a fall after explanation by the research team. This may have had an effect on the cut off scores of each test. Thirdly, this study was adequately powered for geriatric fallers and non‐fallers, but not for sex‐specific analyses with only 15 male participants. In addition, this was a retrospective cohort study. A prospective cohort study would be needed to determine whether the FSST with DT could predict future falls. Moreover, other limitations of this study include demographic limitations and a small sample size. A sample of convenience was used for this study, gathering participants from a local area and surrounding communities. Also, counterbalancing and randomization of test order were not used in this study to control for learning or fatigue effects. Lastly, the number of participants in this study was small and lacked ethnic and racial diversity, limiting the ability to generalize the results to the geriatric population as a whole. The use of a sample of convenience and lack of randomization reduced the external validity and generalizability of this study.

### 4.4 Recommendations for Future Research

While this study has limitations, it provides preliminary evidence supporting further investigation into the use of the FSST with DT conditions to predict falls in individuals over the age of 60. As a functional outcome measure, the FSST has proven utility in assessing mobility in geriatric populations [17]. However, prior studies conducted on non‐geriatric populations may yield less applicable results, as the FSST was originally developed for older adults.

Future research should employ a prospective study design to more accurately assess the predictive validity of the FSST with and without DT components in older adults. Additionally, studies incorporating larger and more diverse samples are needed to improve the generalizability of findings. Research should also explore the appropriateness of a 10‐second cut‐off score versus the commonly cited 15‐second threshold [17] for the FSST without DT. Lastly, to address limitations related to randomization, diversity, and external validity, future studies should consider a multicenter approach to participant recruitment and data collection.

## 5 Conclusion

Findings from this study suggest that the FSST with a motor DT in females and a cognitive DT in both males and females demonstrated higher accuracy in distinguishing older adults with and without a fall history, compared to the FSST without a DT. These results support the potential utility of the FSST with DT conditions as an alternative assessment tool for clinicians aiming to differentiate between fallers and non‐fallers in community‐dwelling older adults.

The FSST with DT is both practical and feasible for clinical implementation, however, standardization in the selection and administration of DT is essential. Evidence‐based tasks such as reciting every other letter of the alphabet or carrying a cup of water should be consistently used to enhance reliability and reproducibility. Lastly, the findings of this study indicate that a 10‐second cut‐off score for the FSST without DT may better discriminate fall risk than the traditional 15‐second threshold [17], which may be too lenient to effectively capture older adult fallers.

## Author Contributions

**Mario Baker:** conceptualization, investigation, writing – original draft, methodology, formal analysis, data curation, supervision, resources, writing – review and editing, project administration, software, validation, visualization. **Liana Burk:** investigation, writing – original draft, writing – review and editing, validation, data curation. **Meredith Gardner:** investigation, writing – original draft, writing – review and editing, validation.

## Funding

The authors received no specific funding for this work.

## Ethics Statement

This study has been approved by the Ethics Committee of The University of Findlay (IRB No. 1662).

## Conflicts of Interest

The authors declare no conflicts of interest.

## Transparency Statement

The lead author Mario Baker affirms that this manuscript is an honest, accurate, and transparent account of the study being reported; that no important aspects of the study have been omitted; and that any discrepancies from the study as planned (and, if relevant, registered) have been explained.

## Supporting information


**Appendix A:** Participant Screening Questionnaire.

**Appendix B:** MMSE.

## Data Availability

The data that support the findings of this study are available on request from the corresponding author. The data are not publicly available due to privacy or ethical restrictions.

## References

[1] D. M. Seabert , J. F. McKenzie , and R. R. Pinger , Mckenzie's: An Introduction to Community and Public Health. Jones and Bartlett Learning, 2022), 230–231. McKenzie's: Tenth Edition.

[2] Older Adult Fall Prevention . Centers for Disease Control. Updated July 14, 2021, accessed July 6, 2022. https://www.cdc.gov/falls/index.html.

[3] Falls . World Health Organization. Updated April 28, 2022, accessed April 28, 2022. https://www.who.int/news-room/fact-sheets/detail/falls (Links to an external site).

[4] P. Mathurapongsakul and A. Siriphorn , “Four Square Step Test With Foam is More Accurate Than Those Without Foam for Discriminating Between Older Adults With and Without Fall History,” Journal of Aging and Physical Activity 26, no. 4 (2018): 624–628. 10.1123/japa.2017-0363. 29431557

[5] A. S. Chiu , R. A. Jean , M. Fleming , and K. Y. Pei , “Recurrent Falls Among Elderly Patients and the Impact of Anticoagulation Therapy,” World Journal of Surgery 42 (2018): 3932–3938, 10.1007/s00268-01847281. 29959494

[6] W. Dite and V. A. Temple , “A Clinical Test of Stepping and Change of Direction to Identify Multiple Falling Older Adults,” Archives of Physical Medicine and Rehabilitation 83, no. 11 (2002): 1566–1571, 10.1053/apmr.2022.35469. 12422327

[7] S. W. Muir‐Hunter and J. E. Wittwer , “Dual‐Task Testing to Predict Falls in Community‐Dwelling Older Adults: A Systematic Review,” Physiotherapy 102, no. 1 (2016): 29–40. 10.1016/j.physio.2015.04.011. 26390824

[8] S. W. Muir , K. Gopaul , and M. M. Montero Odasso , “The Role of Cognitive Impairment in Fall Risk Among Older Adults: A Systematic Review and Meta‐Analysis,” Age and Ageing 41 (2012): 299–308. 22374645 10.1093/ageing/afs012

[9] T. Y. Liu‐Ambrose , M. C. Ashe , P. Graf , B. L. Beattie , and K. M. Khan , “Increased Risk of Falling in Older Community‐Dwelling Women With Mild Cognitive Impairment,” Physical Therapy 88 (2008): 1482–1491. 18820094 10.2522/ptj.20080117 PMC3514550

[10] M. M. Lusardi , S. Fritz , A. Middleton , et al., “Determining Risk of Falls in Community Dwelling Older Adults: A Systematic Review and Meta‐Analysis Using Posttest Probability,” Journal of Geriatric Physical Therapy 40, no. 1 (2017): 1–36, 10.1519/JPT.0000000000000099. 27537070 PMC5158094

[11] P. R. Brustio , E. Rabaglietti , S. Formica , and M. E. Liubicich , “Dual‐Task Training in Older Adults: The Effect of Additional Motor Tasks on Mobility Performance,” Archives of Gerontology and Geriatrics 75 (2018): 119–124, 10.1016/j.archger.2017.12.00311. 29245071

[12] D. A. Henning , E. M. Edwards , M. Ansara , and N. E. Fritz , “Validating the Walking While Talking Test to Measure Motor, Cognitive, and Dual‐Task Performance in Ambulatory Individuals With Multiple Sclerosis,” Multiple Sclerosis and Related Disorders 54 (2021): 103123, 10.1016/j.msard.2021.103123. 34246023

[13] M. N. Lopez , R. A. Charter , B. Mostafavi , L. P. Nibut , and W. E. Smith , “Psychometric Properties of the Folstein Mini‐Mental State Examination,” Assessment 12, no. 2 (2005): 137–144, 10.1177/1073191105275412. 15914716

[14] M. K. Haugeberg Standard and cognitive four square step test (FSST) part II. UND Scholarly Commons, accessed September 5, 2022. https://commons.und.edu/pt-grad/686/.

[15] R. Hoffman and H. Bucholz Standard and cognitive four square step test (FSST). UND Scholarly Commons, accessed September 5, 2022. https://commons.und.edu/pt-grad/679/.

[16] Factors contributing to single and dual task performance of Four‐Square Step tests ‐ full text view . Full Text View ‐ ClinicalTrials.gov, accessed September 2, 2022. https://www.clinicaltrials.gov/ct2/show/NCT03785639.

[17] Four square step test . Shirley Ryan Ability Lab, accessed September 12, 2022. https://www.sralab.org/rehabilitation-measures/four-square-step-test.

