# Supplementary material for: The Four‐Square Step Test With and Without Dual Tasks Among Older Adults With and Without a Fall History: A Retrospective Cohort Study
Source: Health Sci Rep. 2026 Feb 13;9(2):e71820. doi: 10.1002/hsr2.71820 (PMC12904000; doi:10.1002/hsr2.71820)
Supplement: Supplementary file 1 — Appendix A: Participant Screening Questionnaire. [file HSR2-9-e71820-s002.docx]

APPENDIX A

ID#: ___________________________ Participant Screening Questionnaire

The University of Findlay, DPT program

**Participant Screening Questionnaire**

**Part 1. Screen for Inclusion Criteria- Please circle Yes or No to whether or not the following is true about the participant:**

**Yes NO** The participant is at or over the age of 60 years for older adult.

**Yes NO** The participant has the ability to communicate and follow > or = 3 step commands.

**Yes NO** The participant can walk at least 20 feet with or without an assistive device.

**Yes NO** The participant can perform basic ADL without assistance per their report.

**Yes NO** The participant dwells in the community.

**Yes NO** The participant’s Mini-Mental State Examination (MMSE) score was > or = 24

**Part 2. Screen for Exclusion Criteria- Please circle Yes or No to whether or not the following is true about the participant:**

**YES NO** Has a medical condition that would prevent full participation in the balance testing

**YES NO** Has a medical condition that would prevent full participation in the cognitive testing

**YES NO** Currently has pending litigation

**YES NO** Has a vestibular disorders

**YES NO** Has blurred vision

**YES NO** Has diplopia (double vision)

**YES NO** Has a musculoskeletal injury or disease that influences walking ability

**YES NO** Has a neurological disease that impairs walking ability

**YES NO** Has Parkinson’s disease

**YES NO** Had a cerebral vascular accident with residual deficits that impair walking ability

**YES NO** Has a Dementia Diagnosis

**YES NO** Has the participant fallen in the past 12 months?

**Part 3. Demographics- Circle the appropriate answers. ONLY continue if you circled YES to all of part 1 and NO to all of part 2.**

1. Age: _______

1. Gender: Male Female Transgender Other

1. Race: White African American Native American or American Indian Asian or Pacific Islander Other

1. Ethnicity: Not Hispanic or Latino Hispanic or Latino

1. Have you fallen in the past 12 months? YES NO If yes, how many times have you fallen? ___
