# Supplementary figures and images for: The Four‐Square Step Test With and Without Dual Tasks Among Older Adults With and Without a Fall History: A Retrospective Cohort Study
Source: Health Sci Rep. 2026 Feb 13;9(2):e71820. doi: 10.1002/hsr2.71820 (PMC12904000; doi:10.1002/hsr2.71820)

APPENDIX B


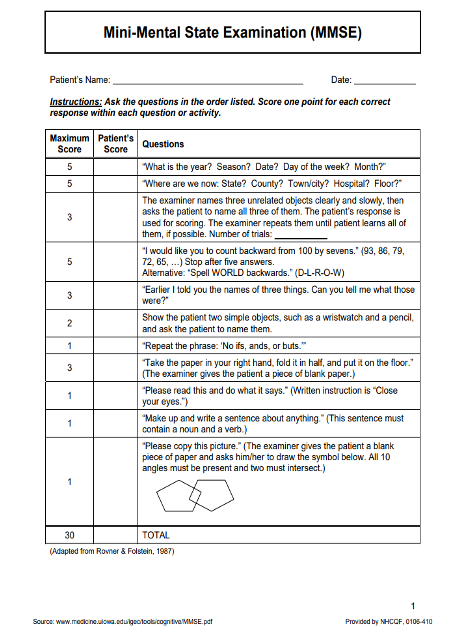

Supplement: Supplementary file 2 — Appendix B: MMSE. [file HSR2-9-e71820-s001.docx]
